# Can ratoon cropping improve resource use efficiencies and profitability of rice in central China?

**DOI:** 10.1016/j.fcr.2019.02.004

**Published:** 2019-03-15

**Authors:** Shen Yuan, Kenneth G. Cassman, Jianliang Huang, Shaobing Peng, Patricio Grassini

**Affiliations:** a National Key Laboratory of Crop Genetic Improvement, MARA Key Laboratory of Crop Ecophysiology and Farming System in the Middle Reaches of the Yangtze River, College of Plant Science and Technology, Huazhong Agricultural University, Wuhan, Hubei, 430070, China; b Department of Agronomy and Horticulture, University of Nebraska-Lincoln, Lincoln, NE, 68583-0915, USA

**Keywords:** Rice, Ratoon rice, Yield, Energy, Environment footprint, Profit

## Abstract

Identifying cropping systems with small global warming potential (GWP) per unit of productivity is important to ensure food security while minimizing environmental footprint. During recent decades, double-season rice (DR) systems in central China have progressively shifted into single-crop, middle-season rice (MR) due to high costs and labor requirements of double-season rice. Ratoon rice (RR) has been proposed as an alternative system that reconciliates both high annual productivity and relatively low costs and labor requirements. Here we used on-farm data collected from 240 farmer fields planted with rice in 2016 to evaluate annual energy balance, environmental impact, and net profit of MR, DR, and RR cropping systems in central China. Energy factors, emission values, and commodity prices obtained from literature and official statistics were used to estimate energy balance, GWP, and economic profit. Average annual yield was 7.7, 15.3. and 13.2 Mg ha^−1^ for MR, DR, and RR systems, respectively. Average total annual energy input (36 GJ ha^−1^), GWP (9783 kg ha^−1^), and production cost (3057 $ ha^−1^) of RR were 35–48% higher than those of MR. However, RR achieved 72–129% higher annual grain yield (13.2 Mg ha^−1^), net energy yield (159 GJ ha^−1^), and net economic return (2330 $ ha^−1^) than MR. Compared with DR, RR produced statistically similar net energy yield while doubling the net economic return, with 32–42% lower energy input, production costs, and GWP. Consequently, RR exhibited significantly higher net energy ratio and benefit-to-cost ratio, and substantially lower yield-scaled GWP than the other two cropping systems. In the context of DR being replaced by MR, our analysis indicated that RR can be a viable option to achieve both high annual productivity and large positive energy balance and profit, while reducing the environmental impact.

## Highlights

• We collected farmer data from middle (MR), double (DR), and ratoon rice (RR) in China.
• We assessed energy balance, carbon footprint, and economic benefit of MR, DR, and RR.
• RR achieved higher annual grain yield, net energy yield, and net economic return than MR.
• RR is an environment-friendly and economically viable alternative for both MR and DR.

## 1 Introduction

Rice is one of the most important staple crops, accounting for *ca.* 21% of global calorie intake (Awika, 2011). China is the largest rice-producing country, with average annual rice production of 210 million metric tons, representing 28% of global rice production (FAO, 2014–2016Food and Agriculture Organization of the United Nations (FAO, 2016FAO, 2014–2016). Major drivers for yield increase in previous decades include increasing usage of commercial fertilizers, pesticides, machinery, and improved cultivars (Cassman, 1999; Fan et al., 2011). However, intensification of agricultural practices has also resulted in negative impacts on the environment and higher production cost (Robertson et al., 2000; Garnett et al., 2013). Hence, there is increasing interest to identify options that can help increase productivity while reducing environmental impact and ensuring profitability.

Middle- (MR) and double-season rice (DR) are the dominant rice systems in central China (Nie and Peng, 2017). DR consists of early- and late-season rice crops, while MR system includes only one rice cycle. Annual total rice yield is typically higher in DR than MR (Chen et al., 2017), but the former system requires more agricultural inputs (*e.g.*, fertilizer, water, labor) and has greater area- and yield-scaled greenhouse gas (GHG) emissions (Feng et al., 2013). Ratio of DR to total rice harvested area has dropped rapidly between mid 1970s and early 2000s because of labor shortage and low benefit-to-cost ratio of DR (Peng et al., 2009; Zhu et al., 2013). Apparently, the transition from DR to MR is likely to continue due to high input and labor costs (Zhu et al., 2013; National Bureau of Statistics of China (NBSC, 2018). In turn, given the lower annual yield in MR *versus* DR, the decline in DR area may eventually reduce total rice production in China. Amid growing national concerns about both rice self-sufficiency and environmental issues, it is relevant to evaluate alternative cropping systems that can meet rice production and environmental goals, while ensuring farmer profitability.

Ratoon rice (RR) is a second rice crop (hereafter called ratoon crop) that sprouts from stem nodes on the rice stubble left behind after the harvest of the first crop (hereafter called main crop) (Jones, 1993). Compared to DR, the RR system does not require the additional labor for transplanting the second rice crop. RR is an old rice-cropping system which has been traditionally practiced and widely planted since 1950 in China (Xu et al., 1988). For example, RR was planted in every province in central China in the 1950s (Xu et al., 1988). RR planted area rapidly increased from 6667 ha in 1988 to 73,000 ha in 1994 in Hubei Province as a result of governmental policy and development of cultivation techniques (Fei et al., 2013; Xu et al., 2015). However, RR area quickly declined thereafter, with only 7000 ha of RR remaining in 2010 in this province. Explanatory factors for the decline in RR area include: (i) lack of suitable rice cultivars for RR systems, (ii) lower and/or unstable yields compared with other rice systems, and (iii) higher labor requirement in RR compared with MR (Li et al., 2014; Xu et al., 2015; Luo, 2016). New rice cultivars with high ratooning ability, together with better crop and water management that allows mechanical harvest of main crop (Xu et al., 2015; Luo, 2016; Tu et al., 2017), have attracted farmers to re-adopt RR in recent years with mechanized RR area of 153,000 ha in Hubei Province in 2017. As a result, mechanized RR system has received renewed attention recently as an alternative cropping system that can meet productivity, economic, and environmental goals (Harrell et al., 2009; Chen et al., 2018). Despite this potential, rigorous quantitative on-farm evaluations of RR systems *versus* dominant MR and DR systems have not been performed for China.

To fulfill this knowledge gap about performance of RR systems in China, the objectives of this study were to compare performance of RR *versus* MR and DR cropping systems in terms of total annual yield, energy, profit, and environmental footprint. For our analysis, we used a large farmer database collected from Hubei Province, central China—a province that accounts for 8% of total rice production in China and includes large portions of cropland under each of the three rice cropping systems (National Bureau of Statistics of China (NBSC, 2018).

## 2 Materials and methods

### 2.1 Site description and data collection

**Fig. 1:**
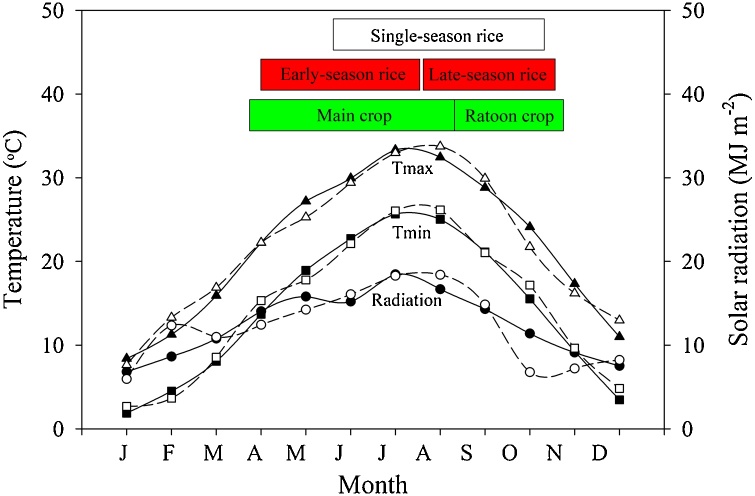
Monthly average incident solar radiation and maximum (Tmax) and minimum temperature (T_min_) based on long-term (1996–2015) weather data (solid line and solid symbols) and in 2016 (dashed line and open symbols) in southeast Hubei Province (29.8°N, 115.6°E). Calendar of middle-season rice (MR), double-season rice (DR), and ratoon rice (RR) cropping systems is shown. Source: National Meteorological Information Center of the China Meteorological Administration (National Meteorological Information Center of the China Meteorological Administration (NMIC, 2017).

We focused on Hubei Province, one of the most important rice producing provinces in China, with total annual rice production of 17 Mt, which represents 8% of total national rice production. The three cropping systems studied here account for 69% (MR), 24% (DR), and 7% (RR) of total land area devoted to rice production in this province (1.7 million ha). Regional weather is classified as humid subtropical with precipitation following a monsoon pattern (Fig. 1). Monthly solar radiation, maximum and minimum temperatures in 2016 were very similar to long-term (1996–2015) averages for these variables. Crop calendars of the three rice-cropping systems are shown in Fig. 1. MR system includes only one rice cycle, which is direct seeded during May and June when warm soil and air temperature ensures proper crop establishment. In contrast, rice is typically transplanted in late March in both DR and RR systems. Low temperature and/or risk of waterlogging early in the season do not allow direct seeding of the main crop in RR and early-season rice in DR because of the high risk of poor seed germination and establishment. An additional constrain for direct seeding in RR is the greater risk of lodging, which negatively affects the growth of regenerated buds (Chen et al., 2018).

For this study, we collected farmer-reported data including grain yield (14% moisture content) and amount of various agricultural inputs used to produce the crops (fertilizer rates, pesticide rates, amount of applied irrigation water, seeding rate, diesel use, electricity use, plastic film, and labor). Data were collected from 240 farmer fields planted with rice in two typical and traditional rice-producing areas in southeast Hubei Province in 2016 through personal interviews. We acknowledge that one year of data may not be sufficient to account for year-to-year variation in weather and its influence on yield, energy balance, and profitability. However, we note that (i) irrigated rice systems usually exhibit high yield stability across years with coefficient of variation typically in the 4–8% range, and (ii) solar radiation and temperature patterns during 2016 were similar to the long-term averages (Fig. 1). The database contained data from 80 fields following each of the three rice-cropping systems (MR, DR, and RR). For each field, data from all crop cycles were collected: one crop cycle in MR, early- and late-season rice in DR and main and ratoon crops in RR. Following Grassini et al. (2014), the degree to which the surveyed data can be considered representative of the population of rice farms in this region was evaluated by comparison of survey yield collected in this study against yield data from official government statistics for the same region in 2016 (National Development and Reform Commission (NDRC, 2017). Because yield data from official government statistics for RR were not available, we only performed the yield comparison between survey data and official statistics for MR and DR.

### 2.2 Calculation of energy balance

Annual energy input and output during the crop growing season were calculated for each of the 240 surveyed fields. Energy inputs include all fossil-fuel energy required for manufacturing, packaging, and transportation of agricultural inputs, including fertilizers, pesticides, seeds, plastic film, machinery, as well as fossil fuel directly used for irrigation pumping and field operations. Human labor was also included in the calculation of energy inputs. Energy inputs were calculated for each field based on the reported input and labor data, and their associated energy equivalents (Supplementary Tables S1 and S2). Energy output was calculated based on farmer-reported grain yield and rice grain energy content (Lal et al., 2015).

Following previous studies (*e.g.*, Grassini and Cassman, 2012), two parameters were used to assess on-farm energy balance: net energy yield (NEY; GJ ha^−1^) and net energy ratio (NER). These parameters were estimated as follows:

(1) NEY=Energy output-Energy input

(2) $$NER=\frac{Energyoutput}{Energyinput}$$

### 2.3 Estimation of environmental footprint

Our inventory of GHG emissions included CO_2_, CH_4_, and N_2_O for each individual field estimated based on farmer-reported inputs, management practices, and associated emission factors. There are three major sources of greenhouse gas (GHG) emissions in rice-production systems: (i) GHGs emissions from production, packaging, and transportation of various agricultural inputs, (ii) N_2_O emissions from nitrogen (N) application either as fertilizer or as manure, and (iii) CH_4_ emission from rice cultivation. Because soil organic matter is typically maintained or increased by intensive, irrigated rice production in lowland areas such as river valleys and flood plains (Bronson et al., 1997; Cassman et al., 1995), we did not include CO_2_ emissions or C sequestration from soil in the inventory.

Annual GHGs from production, storage, and transportation of various agricultural inputs were estimated based on rates of agricultural inputs and associated GHGs emission coefficients (Table S3). Following Grassini and Cassman (2012) and Pittelkow et al. (2014), direct N_2_O emissions from paddy field were estimated using the relationship between N_2_O emission and N surplus reported by van Groenigen et al. (2010), with N surplus calculated as the difference between N input (including inorganic and organic sources) and crop N uptake. The latter was estimated based on farmer-reported grain yield and assumptions on harvest index and grain and straw N concentrations, respectively (Supplementary Table S4). We did not include N inputs from biological N_2_ fixation in the soil-floodwater system, as we did not have direct measurements for the three cropping systems. Total N_2_O emissions were calculated as the sum of direct and indirect N_2_O emissions, with the latter assumed to account for 20% of direct emissions following IPCC (2006). Annual CH_4_ emission from rice cultivation was calculated using a daily emission factor of 1.3 kg CH_4_ ha^−1^ day^−1^ and the approximate duration of the rice crop cycle, and some specific scaling factors associated with water regime and organic amendment (Intergovernmental Panel on Climate Change (IPCC, 2006; Supplementary Table S5). We were aware of recent advances in estimating field-level CH_4_ emissions (Linquist et al., 2018); however, we did not use this approach in our study because (i) not all required data inputs were available (*e.g.*, soil texture and chemical properties) and (ii) this approach has only been validated for temperate regions. In the case of DR and RR, N_2_O, and CH_4_ emissions were calculated for each crop cycle (*i.e.*, early- and late-season in DR and main and ratoon crops in RR) and then summed up for a given cropping system to estimate annual emissions. Paddy fields are not flooded during the non-rice growing period, which drastically reduces CH_4_ emission (Xu et al., 2003). Additionally, Tang et al. (2012) showed that CH_4_ and N_2_O emission fluxes from paddy field during this period are negligible. Therefore, emissions from paddy fields during the non-growing season were not included in our GHG assessment.

Annual GHG emissions, including CO_2_, CH_4_, and N_2_O, were expressed as global warming potential (GWP, kg ha^−1^). For the GWP calculation, we used 100-yr GWP equivalent factors of CO_2_, CH_4_, and N_2_O (1:25:298) as reported by IPCC (2007). Yield-scaled GWP (GWPi; kg Mg^−1^), also known as GHG intensity, was calculated as follows:

(3) $$GWPi=\frac{GWP}{Rice\;yield}$$

### 2.4 Economic analysis

For each field, total gross income, variable costs, and total production costs were calculated based on reported annual input amounts and labor and associated market prices (in US$) around year 2016 (Supplementary Tables S6–S7). Net economic return and benefit-to-cost ratio were calculated as follows:

(4) Net economic return=Total gross income-Total production cost

(5) $$Benefit-to-\cos t\;ratio=\frac{Total\;gross\;income}{Total\;production\;\cos t}$$

Because labor is a key element in determining adoption of new cropping systems, we calculated the net profit-to-labor use ratio (NPL) as follows:

(6) $$Net\;profit-to-laboruse\;ratio=\frac{Net\;economic\;return}{Total\;labor\;input}$$

We note that we did not considered fixed costs in our estimates of production costs and net income. Additionally, we computed the eco-efficiency, which is defined as the ratio of economic benefit to environmental impact:

(7) $$Eco-efficiency=\frac{Net\;economic\;return}{GWP}$$

Data on energy balance, environment footprint, and economic performance of the three rice-cropping systems were subjected to statistical analysis of variance (ANOVA) and means were compared using least significant difference (LSD) test at the 0.05 level of significance.

## 3 Results

### 3.1 Energy balance in farmer rice fields

Average MR yield obtained by farmers in this study was nearly identical to official statistics for Hubei Province (7.7 *versus* 7.8 Mg ha^−1^), while average total annual DR yield (15.3 Mg ha^−1^) was somewhat higher (13%) than average DR yield reported in official statistics (National Development and Reform Commission (NDRC, 2017). Average annual RR yield (13.2 Mg ha^−1^) was within the yield range (12.4-15.7 Mg ha^−1^) reported for the same area in previous studies (Dong et al., 2017). Similarity between yields obtained by farmers in our study (Table 2) and the yields reported in these other independent sources gives confidence that the rice farmers included in our study are representative of rice farming in Hubei Province, and the associated variation in yields and production environments across the three cropping systems.

In all three rice cropping systems N fertilizer and diesel fuel used for mechanized field operations accounted for about 55% of total energy use (*ca.* 40% for N fertilizer and 15% for diesel; Table 1). As expected, inputs were *ca.* two-fold larger in DR *versus* MR because many of the field operations are performed similarly in both crops. Although both DR and RR systems produce two crops of rice, RR systems require substantially less resources on both an area- and a yield-adjusted basis (Table 1). Hence, reduced inputs of N (−28%), diesel fuel use (−29%), irrigation (−18%), and labor (−32%) in RR resulted in higher efficiencies in use of N fertilizer, irrigation water, and labor compared with DR (Table 1).

**Table 1:** Average annual applied inputs (and percentage of total energy input), total fossil-fuel energy input, labor productivity (LP), net profit-to-labor use ratio (NPL), partial factor productivity for N fertilizer (PFP_N_), and irrigation-water productivity (IWP) based on farmer-reported data for middle-season rice (MR), double-season rice (DR), and ratoon rice (RR) cropping systems.

Inputs	Rate (per ha per year)		
	MR	DR	RR
N fertilizer, kg N	226 (39%)	472 (42%)	342 (44%)
P fertilizer, kg P_2_O_5_	139 (14%)	188 (8%)	107 (6%)
K fertilizer, kg K_2_O	197 (13%)	334 (22%)	178 (9%)
Irrigation water, m^3^	2423 (9%)	4004 (8%)	3294 (9%)
Seed, kg	55 (3%)	57 (2%)	26 (1%)
Labor, h	172 (1%)	846 (3%)	572 (3%)
Pesticides, kg a.i.	3.3 (2%)	4.7 (1%)	3.0 (1%)
Machinery, MJ	1448 (6%)	2856 (5%)	2196 (6%)
Fuel use for on-farm operations, l	63 (13%)	130 (14%)	92 (15%)
Total energy input (GJ)	27	53	36
LP, kg grain h^−1^ labor	45	18	23
NPL, $ h^−1^ labor	5.9	1.4	4.1
PFP_N_, kg grain kg^−1^ N fertilizer	34	33	39
IWP, kg grain m^−3^ water irrigation	3.2	3.8	4.0

Pesticides were calculated as the total of insecticide, herbicide, and fungicide used in rice production.

a.i.: active ingredient.

**Table 2:** Minimum, maximum, 25^th^ and 75^th^ percentiles (P25 and P75, respectively), mean, and coefficient of variation (CV, in %) for annual productivity, energy balance, environmental footprint, and economic indicators for the three rice cropping systems.

Variable	Minimum	P25	Mean	P75	Maximum	CV
Middle rice						
Grain yield	7.0	7.5	7.7	8.0	8.6	5
Energy input	23	26	27	27	30	6
Energy output	103	110	113	117	127	5
NEY	76	84	87	89	97	5
NER	3.9	4.1	4.3	4.4	4.7	4
GWP	6343	6934	7211	7466	8189	5
GWPi	858	912	936	961	1018	4
Total production cost	1688	1941	2068	2186	2484	8
Net economic return	774	927	1019	1108	1251	12
Benefit-to-cost ratio	1.3	1.4	1.5	1.6	1.7	6
Eco-efficiency	107	130	142	155	194	14

Double season rice						
Grain yield	13.9	14.7	15.3	15.7	16.5	4
Energy input	44	50	53	55	59	6
Energy output	204	216	224	231	243	4
NEY	155	165	172	179	189	5
NER	3.7	4.1	4.3	4.4	4.9	5
GWP	14908	16400	16835	17345	18672	4
GWPi	1019	1078	1104	1132	1196	4
Total production cost	4595	4954	5088	5230	5597	4
Net economic return	900	1010	1143	1260	1500	13
Benefit-to-cost ratio	1.2	1.2	1.2	1.3	1.3	2
Eco-efficiency	52	61	68	75	87	13

Ratoon rice						
Grain yield	11.8	12.8	13.2	13.7	14.3	4
Energy input	34	35	36	37	39	3
Energy output	174	189	195	201	210	4
NEY	141	153	159	164	171	4
NER	5.1	5.3	5.4	5.5	5.8	3
GWP	9254	9599	9783	9940	10363	2
GWPi	715	728	740	749	781	2
Total production cost	2782	2970	3057	3146	3515	4
Net economic return	1802	2190	2330	2459	2774	8
Benefit-to-cost ratio	1.6	1.7	1.8	1.8	2.0	4
Eco-efficiency	192	228	238	247	271	6

Units: grain yield (Mg ha^−1^), energy inputs and outputs (GJ ha^−1^), and NEY (GJ ha^−1^), GWP (kg ha^−1^), GWPi (kg Mg^−1^), NER and benefit-to-cost ratio (unitless), total production cost and net economic return ($ ha^−1^) and eco-efficiency ($ Mg^−1^).

As expected, DR and RR had higher annual yield, energy output, and NEY than MR because of higher harvest intensity, with grain yield averaging 7.7 (MR), 15.3 (DR;), and 13.2 Mg ha^−1^ (RR) (Table 2). In DR, grain yield averaged 7.2 and 8.1 Mg ha^−1^ for early- and late-season rice, respectively. Main and ratoon crops in RR averaged respective 7.5 and 5.7 Mg ha^−1^. Consistent with differences in applied inputs across cropping systems shown in Table 1, DR had higher annual energy inputs (53 GJ ha^−1^) compared with RR (36 GJ ha^−1^) and MR (27 GJ ha^−1^) (*P* < 0.01).

**Fig. 2:**
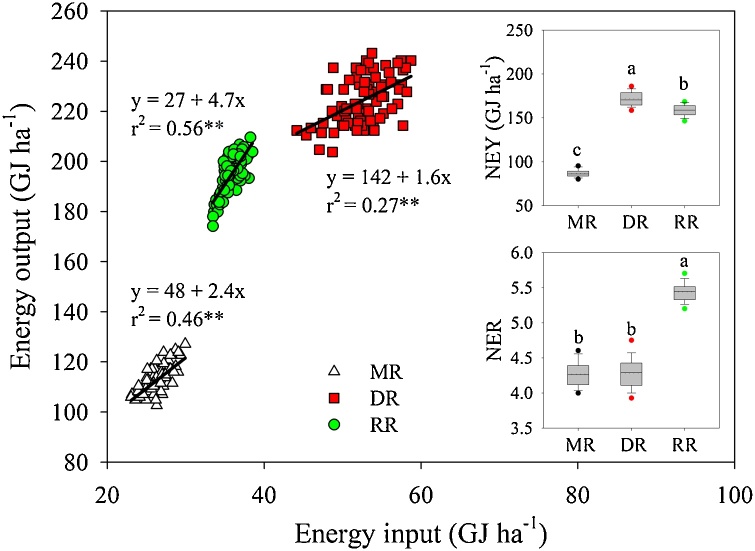
Annual grain energy output *versus* fossil-fuel energy input for middle-season rice (MR), double-season rice (DR), and ratoon rice (RR) farmer fields. Separate linear regressions were fitted for each cropping system; parameters and coefficients of determination (r^2^) are shown. Asterisks indicate that the slope of the fitted linear regression was statistically different from zero (P < 0.01). Insets show boxplots for net energy yield (NEY) and net energy ratio (NER), with solid and dotted lines inside the box indicating median and mean values, respectively, box boundaries denoting the upper and lower quartiles, whiskers representing the 10th and 90th percentiles, and the circles representing the 95th and 5th percentiles. Letters indicate statistically significant differences (LSD test, P < 0.01). Overall regression for the pooled data including the three cropping systems: y = 3.9 x + 28 (*r^2^*= 0.70; P<0.01)

Annual grain energy output was closely related to fossil-fuel energy input, with the latter explaining both variation among cropping systems as well as field-to-field variation within cropping systems (Fig. 2). Indeed, regression analysis indicated a statistically significant relationship between yield and energy inputs for all cropping systems and for the pooled data. However, DR exhibited a weaker energy output-input relationship ( *r^2^* =0.27 *versus* 0.46 and 0.56 in MR and RR, respectively) and the range of energy inputs across DR fields *ca.* 3-fold larger compared with MR and RR fields (Table 2, Fig. 2).

The RR system attained higher NER than MR and DR (5.4 *versus* 4.3) due to a combination of higher annual productivity (compared with MR) and smaller energy input (compared with DR) (Table 2; Fig. 2). Likewise, NEY in RR was 83% higher than in MR and only 8% lower compared with DR. To summarize, RR outperformed MR in relation with yield, NEY, and NER, achieving lower (13%) annual yields compared with DR but using substantially less (32%) energy inputs. Finally, field-to-field variation in yield, NER, and NEY was relatively low (<5%) and very similar across the three cropping systems (Table 2).

### 3.2 Environmental footprint across rice systems

**Fig. 3:**
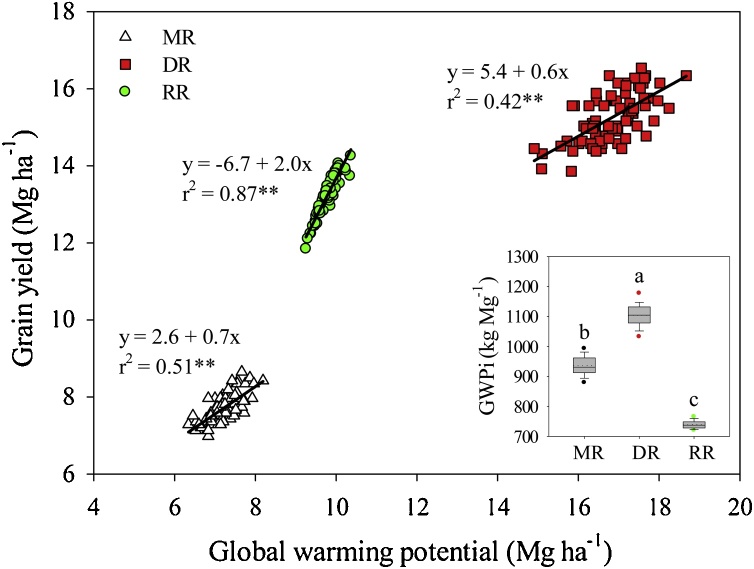
Annual grain yield *versus* global warming potential for middle-season rice (MR), double-season rice (DR), and ratoon rice (RR) systems. Separate linear regressions were fitted for each cropping system; parameters and coefficient of determination (r^2^) are shown. Asterisks indicate the slope of the fitted linear regression was statistically different from zero (P < 0.01). Inset shows boxplot of global warming potential intensity (GWPi) for each cropping systems (see description of boxplots in caption to Fig. 2). Letters indicate statistically significant differences (LSD test, P < 0.01).

Annual GWP per unit area was different among the three cropping systems, decreasing in this order: DR-RR-MR (Table 2, Fig. 3). High GWP in DR and RR was primarily due to larger emissions associated with longer rice growing period and larger input amounts compared with MR (Fig. 1, Table 2). Indeed, the relationship between grain yield and GWP indicate increased environmental footprint with increasing yield as a results of larger input application (Fig. 3). Additionally, Fig. 2, Fig. 3 showed that the relationship between grain yield and GWP is similar in form and goodness of fit to the relationship between yield and energy input because fossil fuel input has such a large influence on the amount of GHG emissions. However, when GWP was expressed per unit of grain produced, RR exhibited the lowest GWPi among the three cropping systems, which was 33% and 21% smaller compared with DR and MR systems, respectively (*P* < 0.01) (Table 2; Fig. 3). Smaller GWPi in RR was due to smaller N fertilizer and fuel inputs compared with DR and higher annual productivity compared with MR (Table 1, Table 2).

**Fig. 4:**
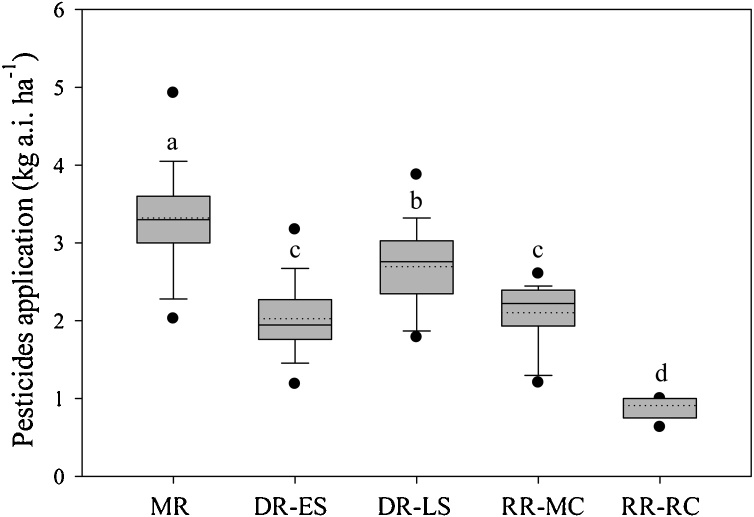
Box plots for applied pesticide inputs in middle-season rice (MR), early- (DR-ES) and late-season rice (DR-LS) of double rice system, and main (RR-MC) and ratoon crops (RR-RC) of ratoon rice system. See description of boxplots in caption to Fig. 2. Letters indicate statistically significant differences (LSD test, P < 0.01).

On an annual basis, DR received the largest pesticide inputs, followed by MR and RR (*P* < 0.01) (Table 1, Fig. 4). However, on a cycle-basis, the MR system received the largest pesticide input. There were large differences in pesticide inputs between crop cycles within the DR and RR systems (Fig. 4). For example, applied pesticide was 33% larger in late *versus* early-season rice in the DR system (*P* < 0.01). Likewise, the ratoon crop received 57% smaller pesticide input compared with the main crop in the RR system (*P* < 0.01).

### 3.3 Labor requirements and economic performance

Labor was 5-fold and 3.3-fold larger in DR and RR compared with MR, with RR using 32% less labor compared with DR (Table 1). As explained previously, MR is direct-seeded while transplanted rice is dominant in DR and RR systems; we note that each transplanting requires 247 h ha^−1^ of labor while direct seeding only takes 28 h ha^−1^. Hence, higher labor usage in DR and RR compared with MR is because of (i) more field operations *and* (ii) higher labor requirement for transplanting compared with direct seeding. Consequently, MR obtained the highest NPL (5.9 $ h^−1^), followed by RR (4.1 $ h^−1^) and DR (1.4 $ h^−1^) (Table 1).

**Fig. 5:**
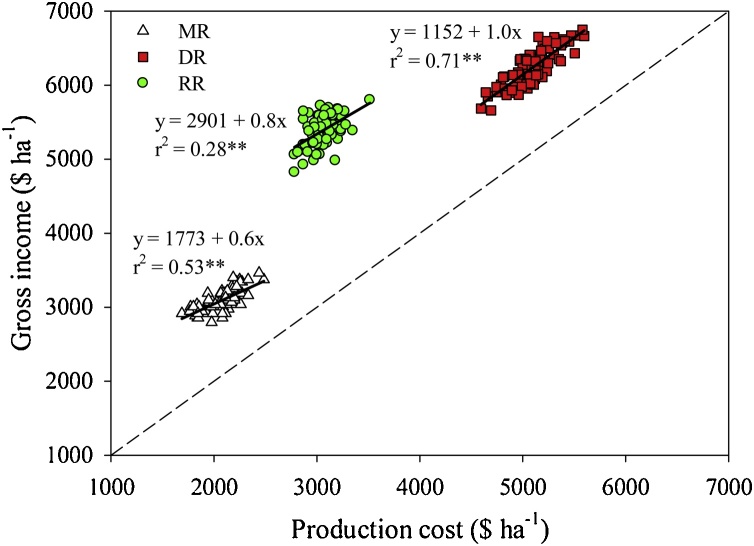
Annual gross income *versus* production cost for middle-season rice (MR), double-season rice (DR), and ratoon rice (RR) cropping systems. Separate linear regressions were fitted for each cropping system; parameters and coefficient of determination (r^2^) are shown. The dashed line indicates x = y (*i.e.*, when gross income equal variable costs). Asterisks indicate that the slope of the fitted linear regression was statistically different from zero (P < 0.01).

**Fig. 6:**
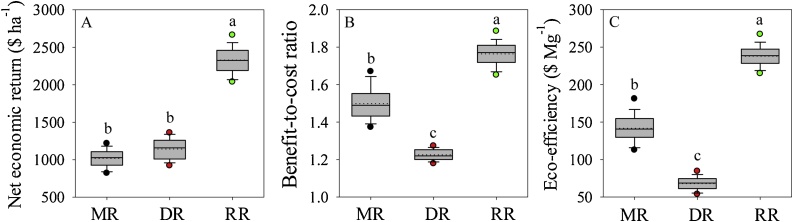
Boxplots of net economic return (A), benefit-to-cost ratio (B), and eco-efficiency (C) for middle-season rice (MR), double-season rice (DR), and ratoon rice (RR) cropping systems. See description of boxplots in caption to Fig. 2. Letters indicate statistically significant differences (LSD test, P < 0.01).

Annual production costs were notably higher in DR compared with MR and RR (Fig. 5, Fig. 6). The difference in total production cost among the three cropping systems was mainly attributable to differences in labor, fertilizers, and fossil fuel inputs (Table 1). Although DR attained the highest gross income, there was a weak relationship between gross and net income ( *r^2^* = 0.12, *P* < 0.01). Indeed, RR was the most profitable rice system, more than doubling annual net profit compared with the other two systems (Fig. 6). These findings were also consistent with differences in benefit-to-cost ratio and eco-efficiency among the three systems, indicating that RR fields attained larger economic benefit per unit of production cost and per unit of environment footprint compared with the two traditional rice systems.

## 4 Discussion

We performed here an on-farm assessment of annual productivity, energy balance, environmental footprint, and economic performance between RR and dominant MR and DR systems. Findings from this study showed that RR exhibited higher profit per unit of production cost and per unit of environment footprint compared with the dominant MR and DR systems, which may explain the gradual transition from MR and DR to RR occurred in central China (Xu et al., 2015; Dong et al., 2017). Furthermore, given growing public negative perception about use of pesticides in agriculture, higher farm-gate purchase price is expected for the ratoon crop because of lower pesticide inputs compared with other rice cycles, which will further increase the profitability of RR system in the future. Annual yield in RR is 13% lower compared with DR; hence, at a broader scale, switching from DR to RR will likely imply an equivalent reduction in regional rice production. However, we note this yield penalty is much smaller compared with the 50% yield reduction that would occur when shifting from DR to MR. Moreover, if current area planted with MR shifts into RR, this can potentially compensate the production reduction associated with the transition from DR to RR. For example, shifting DR and MR area in Hubei Province into RR cultivation would lead into an overall 34% increase in total rice production. We note that this scenario is optimistic as it assumes that all MR area can shift into RR, which is unrealistic given the larger irrigation water requirements and accumulated thermal time in RR *versus* MR which makes RR inviable in areas where water supply and cumulative temperature are limiting (Sun, 1991).

The RR system achieved higher efficiency in the use of key agricultural inputs such as N fertilizer and water compared with MR and DR systems. Likewise, RR exhibited higher labor productivity compared with DR. This is relevant in the context of low input-use efficiency and labor shortage in rice-based systems in China and other parts of Asia (Peng et al., 2009; Yuan et al., 2017). Higher input-efficiency in the RR system lead to smaller GHG intensity and higher profit, compared with the other two dominant rice systems. Findings derived from this study are relevant for other lowland rice systems worldwide, especially in regions with a length of growing season that allows cultivation of RR and with reasonable degree of mechanization and access to extension services, markets, and inputs such as many tropical and subtropical areas in southeast and south Asia.

There are still constrains for RR adoption including (i) lack of explicit breeding for improved cultivars for RR systems, (ii) limited knowledge on the high-yielding and yield stability of ratoon crop and best agronomic practices to improve productivity and input-use efficiency; and (iii) increased risk associated with insect and diseases pressures (Xu et al., 2015; Negalur et al., 2017; Chen et al., 2018). Likewise, we note that labor requirement in RR is at present *ca.* 3-fold higher than MR, which may constrain RR adoption. A directed-seeded RR with lodging-resistant cultivar might be a promising rice farming system to further reduce labor input and increase input-use efficiency together (Chen et al., 2018). Still, findings of this study clearly show that RR can be a viable alternative for farmers shifting from DR to MR and it can also help to intensify current MR systems as long as water and cumulative temperature are not limiting.

## 5 Conclusions

This study assessed energy balance, economic benefit, and environmental footprint of three rice systems in China. Except for GWP and labor productivity, the RR system outperformed MR in all variables, including annual grain yield, while it produced (13%) lower yield than DR but using 32% less total energy input and with 40% lower production costs, resulting in smaller GWP, higher NER, and higher economic profit than DR. Overall, results show that RR is a viable alternative for both MR and DR farmers. The impact on regional rice production would depend on the degree to which DR area shifts into RR *versus* MR area that shifts into RR, with the latter depending upon water availability and cumulative temperature. Findings from this analysis may provide valuable insights to other agricultural provinces in China and other rice producing regions in the world with similar biophysical and socio-economic situation to improve current rice production in term of grain output, environmental footprint, and farm profit.
