# Supplementary material for: Can ratoon cropping improve resource use efficiencies and profitability of rice in central China?
Source: Field Crops Res. 2019 Mar 15;234:66–72. doi: 10.1016/j.fcr.2019.02.004 (PMC6472545; doi:10.1016/j.fcr.2019.02.004)
Supplement: Supplementary file 1 [file mmc1.docx]

**Supplementary Materials**

**Supplementary Table S1**. Energy equivalents of various inputs in rice production.

| Particulars | Unit | Energy equivalent (MJ unit^-1^) | References |
| --- | --- | --- | --- |
| Machinery | kg | 68.4 | Pellegrini and Fernández, 2018 |
| Labor | h | 1.96 | Singh et al., 2008 |
| Diesel | l | 56.31 | Erdal et al., 2007 |
| Urea | kg | 26.5 | IFA, 2009 |
| Ammonium bicarbonate | kg | 49.3 | IFA, 2009 |
| Superphosphate | kg | 3.1 | IFA, 2009 |
| Potash muriate | kg | 5.8 | IFA, 2009 |
| N-P-K fertilizer | kg | 10.3 | IFA, 2009 |
| Water | m^3^ | 1.02 | Yuan and Peng, 2017 |
| Insecticide | kg | 101.2 | Yang and Chen, 2012 |
| Herbicide | kg | 238 | Yang and Chen, 2012 |
| Fungicide | kg | 216 | Yang and Chen, 2012 |
| Rice seed | kg | 14.7 | Tabar et al., 2010; Lal et al., 2015 |
| Plastic film | kg | 158 | Boustead, 2003 |
| Electricity | kWh | 3.6 | CPGC, 2007 |

**Supplementary Table S2**. Description of parameters needed for estimating energy inputs and GHG emissions associated with machinery.

| Implements | Unit weight (kg) | Fuel consumption (h^-1^) | Operational life  (h) | Field working efficiency  (%) | Power source |
| --- | --- | --- | --- | --- | --- |
| Plowing machine | 1215 | 2.1 l | 2500 | 65 | Diesel |
| Harvester | 2600 | 4.0 l | 3500 | 55 | Diesel |
| Sprayer | 6.5 | 35 W | 750 | 80 | Electricity |

*Estimation of machinery energy inputs*

Energy input derived from machinery use was computed as follows:

$$Energy_{mach}=\frac{WH\times MTR\times WM}{OL\times Eff}$$

wherein Energy_mach_ is the energy input from machinery (MJ ha^-1^). WH, MTR, WM, OL, and Eff are working hour of machinery in the field; energy used to manufacture, transport, and repair the machinery; weight of machinery; total operational life of machinery; and field working efficiency, respectively.

**Supplementary Table S3**. Emission factors used for estimating greenhouse gases emissions from manufacturing, packaging, and transportation of agricultural inputs.

| Particulars | Unit | Emission factor (CO_2_-eq kg unit^-1^) | References |
| --- | --- | --- | --- |
| Machinery | MJ | 0.071 | Dyer and Desjardins, 2006 |
| Diesel | l | 2.75 | Cheng et al., 2011 |
| Urea | kg | 2.51 | Brentrup et al., 2016 |
| Ammonium bicarbonate | kg | 1.67 | Zhang et al., 2013 |
| Superphosphate | kg | 0.17 | IFA, 2009 |
| Muriate of potash | kg | 0.23 | Brentrup et al., 2016 |
| N-P-K fertilizer | kg | 1.73 | Brentrup et al., 2016 |
| Insecticide | kg | 5.1 | Lal, 2004 |
| Herbicide | kg | 6.3 | Lal, 2004 |
| Fungicide | kg | 3.9 | Lal, 2004 |
| Rice seed | kg | 0.78 | Guo et al., 2017 |
| Plastic film | kg | 1.47 | Narita et al., 2002 |
| Electricity | KWh | 1.12 | Zhang et al., 2013 |

**Supplementary Table S4**. Harvest index and tissue N concentration for middle-season rice, double-season rice, and ratoon rice used to calculate soil N_2_O emissions of paddy field. Values were computed based on Wang (2016), Liu (2017), Yuan et al. (2017), and Wang (unpublished data).

| Cropping system | Crop cycle | Harvest index | Grain N concentration  (%) | Straw N concentration  (%) |
| --- | --- | --- | --- | --- |
| Middle-season rice | | 0.52 | 1.35 | 0.82 |
| Double-season rice | Early-season | 0.56 | 1.00 | 0.84 |
|  | Late-season | 0.56 | 0.98 | 0.87 |
| Ratoon rice | Main crop | 0.56 | 1.34 | 1.53 |
|  | Ratoon crop | 0.54 | 1.11 | 0.72 |

**Supplementary Table S5**. Values of scaling factors used to estimate methane emission from rice cultivation (IPCC, 2006).

| Cropping system | Crop cycle | SFw | SFp | CFOA |
| --- | --- | --- | --- | --- |
| Middle-season rice | | 0.60 | 0.68 | 0.29 |
| Double-season rice | Early-season | 0.60 | 1.00 | 0.29 |
|  | Late-season | 0.52 | 1.00 | 1.00 |
| Ratoon rice | Main crop | 0.60 | 1.00 | 0.29 |
|  | Ratoon crop | 0.60 | 1.00 | 0.00 |

SFw is the scaling factor that accounts for differences in water regime during the growing season; SFp is the scaling factor that accounts for differences in water regime during the non-growing season; CFOA is the conversion factor for organic amendments (IPCC, 2006).

**Supplementary Table S6.** Unit prices of the various inputs used to evaluate economic performance in three rice systems in China. Data were retrieved from the Hubei Agricultural Technology Extension and Service Station.

| Particulars | Unit | Price (US$ unit^-1^) |
| --- | --- | --- |
| Machinery | h | 8.28 |
| Labor | h | 2.71 |
| Diesel | l | 2.71 |
| Urea | kg | 0.29 |
| Ammonium bicarbonate | kg | 0.08 |
| Superphosphate | kg | 0.10 |
| Muriate of potash | kg | 0.45 |
| N-P-K fertilizer | kg | 0.78 |
| Insecticide | kg | 72.26 |
| Herbicide | kg | 120.44 |
| Fungicide | kg | 316.16 |
| Rice seed | kg | 7.53 |
| Plastic film | kg | 0.45 |
| Electricity | kWh | 0.08 |

**Supplementary Table S7.** Rice grain prices used to calculate economic performance of three rice systems. Data were retrieved from the Hubei Agricultural Technology Extension and Service Station.

| Cropping system | Crop cycle | Unit | Price (US$ unit^-1^) |
| --- | --- | --- | --- |
| Middle-season rice |  | kg | 0.40 |
| Double-season rice | Early-season | kg | 0.40 |
|  | Late-season | kg | 0.42 |
| Ratoon rice | Main crop | kg | 0.40 |
|  | Ratoon crop | kg | 0.42 |

**References – Supplementary materials**

Boustead, I., 2003. Polymers and energy. In: Andrady, A.L., ed. Plastics and the Environment. John Wiley & Sons; pp. 123-133.

Brentrup, F., Hoxha, A., Christensen, B., 2016. Carbon footprint analysis of mineral fertilizer production in Europe and other world regions. In: Proc of 10th International Conference on Life Cycle Assessment of Food 2016, Dublin UCD pp.482-490.

Central People’s Government of China (CPGC), 2007. National plan for coping with climate change. http://www.gov.cn/gongbao/content/2007/content_678918.htm (in Chinese).

Cheng, K., Pan, G., Smith, P., Luo, T., Li, L., Zheng, J., Zhang, X., Han, X., Yan, M., 2011. Carbon footprint of China's crop production-An estimation using agro-statistics data over 1993-2007. Agr. Ecosyst. Environ. 142, 231-237.

Dyer, J.A., Desjardins, R.L., 2006. Carbon dioxide emissions associated with the manufacturing of tractors and farm machinery in Canada. Biosyst. Eng. 93, 107-118.

Erdal, G., Esengün, K., Erdal, H., Gündüz, O., 2007. Energy use and economical analysis of sugar beet production in Tokat Province of Turkey. Energy 32, 35-41.

Guo, J., Song, Z., Zhu, Y., Wei, W., Li, S., Yu, Y., 2017. The characteristics of yield-scaled methane emission from paddy field in recent 35-year in China: A meta-analysis. J. Clean. Prod. 161, 1044-1050.

International Fertilizer Industry Association (IFA), 2009. Fertilizers, climate change and enhancing agricultural productivity sustainably. Paris, France: International Fertilizer Industry Association.

Intergovernmental Panel on Climate Change (IPCC), 2006. 2006 IPCC Guidelines for National Greenhouse Gas Inventories. Institute for Global Environmental Strategies, Hayama, Kanagawa, Japan.

Lal, B., Panda. B.B., Gautam, P., Raja, R., Singh, T., Mohanty, S., Shahid, M., Tripathi, R., Kumar, A., Nayak, A.K., 2015. Input-output energy analysis of rainfed rice-based cropping systems in eastern India. Agron. J. 107, 1750-1756.

Lal, R., 2004. Carbon emission from farm operations. Environ. Int. 30, 981-990.

Liu, H.Y., 2017. Physiological mechanism of high yield and high resources use efficiency of dry seeded rice under different water managements. Ph.D. thesis. Huazhong Agricultural University, Wuhan (in Chinese with English abstract).

Narita, N., Sagisaka, M., Inaba, A., 2002. Life cycle inventory analysis of CO_2_ emissions manufacturing commodity plastics in Japan. Int. J. Life Cycle Assess. 7, 277-282.

Pellegrini, P., Fernández, R.J., 2018. Crop intensification, land use, and on-farm energy-use efficiency during the worldwide spread of the green revolution. Proc. Natl. Acad. Sci. USA 115, 2335-2340.

Singh, K.P., Prakash, V., Srinivas, K., Srivastva, A.K., 2008. Effect of tillage management on energy use efficiency and economics of soybean (*Glycine max*) based cropping systems under the rainfed condition in North-West Himalayan region. Soil Till. Res. 100, 78-82.

Tabar, I.B., Keyhani, A., Rafiee, S., 2010. Energy balance in Iran's agronomy (1990-2006). Renew. Sust. Energy Rev. 14, 849-855.

Wang, D.P., 2018. Effects of integrated crop management, climatic conditions, and soil fertility on dry matter production and yield formation of double-season rice and the related mechanism. Ph.D. thesis. Huazhong Agricultural University, Wuhan (in Chinese with English abstract).

Yang, Q., Chen, G.Q., 2012. Nonrenewable energy cost of corn-ethanol in China. Energy Pol. 41, 340-347.

Yuan, S., Nie, L., Wang, F., Huang, J., Peng, S., 2017. Agronomic performance of inbred and hybrid rice cultivars under simplified and reduced-input practices. Field Crops Res. 210, 129-135.

Yuan, S., Peng, S., 2017. Input-output energy analysis of rice production in different crop management practices in central China. Energy 141,1124-1132.

Zhang, W., Dou, Z., He, P., Ju, X., Powlson, D., Chadwick, D., Norse, D., Lu, Y., Zhang, Y., Wu, L., Chen, X., Cassman, K.G., Zhang, F., 2013. New technologies reduce greenhouse gas emissions from nitrogenous fertilizer in China. Proc. Natl. Acad. Sci. USA 110, 8375-8380.
